# Electroporation-Mediated Delivery of Cas9 Ribonucleoproteins Results in High Levels of Gene Editing in Primary Hepatocytes

**DOI:** 10.1089/crispr.2021.0134

**Published:** 2022-06-08

**Authors:** Tanner Rathbone, Ilayda Ates, Lawrence Fernando, Ethan Addlestone, Ciaran M. Lee, Vincent P. Richards, Renee N. Cottle

**Affiliations:** ^1^ Department of Bioengineering, University College Cork, Cork, Ireland.; ^2^ APC Microbiome Ireland, University College Cork, Cork, Ireland.; ^3^ Department of Biological Sciences, Clemson University, Clemson, South Carolina.

## Abstract

Adeno-associated virus vectors are the most used delivery method for liver-directed gene editing. Still, they are associated with significant disadvantages that can compromise the safety and efficacy of therapies. Here, we investigate the effects of electroporating CRISPR-Cas9 as mRNA and ribonucleoproteins (RNPs) into primary hepatocytes regarding on-target activity, specificity, and cell viability. We observed a transfection efficiency of >60% and on-target insertions/deletions (indels) of up to 95% in primary mouse hepatocytes electroporated with Cas9 RNPs targeting *Hpd*, the gene encoding hydroxyphenylpyruvate dioxygenase. In primary human hepatocytes, we observed on-target indels of 52.4% with Cas9 RNPs and >65% viability after electroporation. These results establish the impact of using electroporation to deliver Cas9 RNPs into primary hepatocytes as a highly efficient and potentially safe approach for therapeutic liver-directed gene editing and the production of liver disease models.

## Introduction

Therapeutic gene editing has recently advanced to clinical trials for inherited metabolic diseases (IMDs) of the liver.^1^ Despite the unprecedented capacity to induce double-stranded breaks at nearly any site in the genome, a significant barrier for liver-directed gene-editing therapies using CRISPR-Cas9 nucleases is the absence of safe and effective protocols for delivering CRISPR components into primary hepatocytes.

Adeno-associated virus (AAVs) vectors are the most commonly used delivery method for CRISPR-Cas9 due to the availability of well-established protocols and high transduction efficiency: Many preclinical studies have reported successful CRISPR-Cas9-mediated gene editing using AAVs in animal models of human IMDs of the liver, including phenylketonuria,^2^ ornithine transcarbamylase deficiency,^3^ and familial hypercholesterolemia.^4^

Diverse AAV serotypes show strong liver tropism and promising outcomes in clinical trials,^5,6^ making AAVs a practical candidate for liver-directed gene editing. However, AAVs have immunogenicity risks stemming from preexisting immunity due to prior exposure to the wild-type (WT) virus, which results in the loss of transduced hepatocytes and therapeutic failure.^6–14^ Further, AAVs have the potential to integrate into on- and off-target Cas9 sites.^15,16^ Insertional mutagenesis of AAV vectors caused hepatocellular carcinoma in neonatal mice.^17,18^ Because DNA cargo delivered by AAVs persist as stable episomes, there are concerns that persistent Cas9 expression may occur, resulting in increased off-target activity and genotoxicity.^19^

In addition, Cas9 immunity is highly prevalent, with up to 78% of humans having anti-Cas9 IgG antibodies and Cas9-specific T cells.^20,21^ Anti-Cas9 cytotoxic T cells are particularly problematic. They can eliminate any cell presenting Cas9 peptides on their major histocompatibility complex class I surface molecules. In the study by Li *et al*., AAV delivery of CRISPR-Cas9 in mice immunized against Cas9 led to cytotoxic T-cell responses and the elimination of gene-edited hepatocytes.^22^ Self-deleting AAVs were proposed to overcome Cas9 immunity^23^ but cannot entirely remove Cas9 and would require short-term immunosuppression therapy to avoid an immune response against the AAV capsid. Thus, immunogenicity complications pose a significant obstacle to clinical translation of AAV for gene editing.

Nonviral methods have the potential to address challenges associated with AAV-mediated delivery of gene-editing reagents into target cells.^1^ Nonviral approaches allow for the possible delivery of transient, potent forms of Cas9, such as mRNA and ribonucleoproteins (RNPs), which exist for shorter periods than plasmid DNA and are associated with higher gene-editing specificity.^24–26^ Electroporation is a nonviral approach that involves utilizing high-voltage currents to permeabilize membranes to deliver biomolecules into cells. Current CRISPR-Cas9 clinical trials use electroporation for gene editing T cells and CD34^+^ hematopoietic stem and progenitor cells (HSPCs).^27–29^ The advantage of electroporation is its potential to be used in a wide array of cell types at all cell-cycle stages.^1,30^

Electroporation is particularly powerful for CRISPR-Cas9-mediated gene editing in target cells. It is amenable to delivery of synthetic chemically modified single-guide RNA (sgRNA), along with Cas9 mRNA or proteins to improve on-target gene editing and to reduce off-target activity.^26,31^ In contrast to *in vivo* gene-editing approaches, *ex vivo* gene editing using electroporation is safer because the gene editing is limited to the intended target cell type and not the whole organism. However, *ex vivo* gene editing is associated with more processing steps: cell isolation from the host, gene editing, and transplantation.

In recent preclinical studies, *ex vivo* gene editing in hepatocytes followed by transplantation corrected a mouse model of hereditary tyrosinemia type I (HTI). HTI is characterized by homozygous loss-of-function mutations in the gene encoding fumarylacetoacetate hydrolase (Fah). *Fah^–/–^* mice were rescued by transplanting gene-corrected hepatocytes co-transduced *ex vivo* using lentiviral vectors containing *Fah*-aiming CRISPR-Cas9 and AAV vector containing a donor template.^32^ In a separate study, hepatocytes transduced utilizing a pair of AAVs to deliver *Fah*-CRISPR-Cas9 and donor template followed by culturing for up to 72 h were capable of engraftment *in vivo* and prevented liver failure.^33^ These studies demonstrate the feasibility of *ex vivo* gene editing in hepatocytes to treat IMDs of the liver.

The shortage of disease models represents an additional major challenge for developing novel therapies for IMDs of the liver. Isolated primary human hepatocytes are the gold standard for drug development studies for treating liver disease, but disease-specific hepatocytes are limited. Induced pluripotent stem cells (iPSCs) differentiated into hepatocyte-like cells are an alternative to primary hepatocytes for disease modeling and drug discovery studies.^34^ The advantage of iPSC-derived hepatocyte-like cells is that the precursor cells can be edited using CRISPR-Cas9 and screened to identify disease-specific mutations for high-throughput drug screens. The disadvantage of iPSC-hepatocyte-like cells is that they provide insufficient levels of engraftment to make disease models.^35^

An alternative disease model development strategy for IMDs of the liver involves *ex vivo* gene editing in primary human hepatocytes followed by transplantation into FRGN mice (*Fah^−/−^*/*Rag2^−/−^*/*Il2rg^−/−^* on the NOD-strain background) that supports the replacement of the native liver by human hepatocytes.^36^ Electroporation is an attractive delivery method for generating disease models because it allows for rapid *ex vivo* delivery of DNA, RNA, and proteins into primary hepatocytes. The electroporated gene-edited cells can subsequently be transplanted into FRGN mice to generate novel models of liver disease.

In this study, we demonstrate the use of electroporation to deliver CRISPR-Cas9 into human and mouse hepatocytes and evaluate Cas9 on-target activity, specificity, and cell viability. To show proof-of-principle gene editing for an IMD of the liver, we designed CRISPR-Cas9 targeting hydroxyphenylpyruvate dioxygenase (Hpd), a therapeutic target for HTI.^37^ Our study results are the first to demonstrate electroporation-mediated delivery of CRISPR-Cas9 into primary hepatocytes and show high levels of gene editing.

## Methods

### Hepatocyte isolation

Animal care and experiments were all under the guidelines and approved protocols of the Institutional Animal Care and Use Committee at Clemson University. Hepatocytes were isolated from anesthetized male C57BL/6J mice 8–10 weeks old using a three-step perfusion procedure as described in Grompe *et al*.^38^

Briefly, the inferior vena cava was cannulated and perfused with three solutions. The final solution to complete *in situ* digestion consisted of EBSS with Ca^2+/^Mg^2+^ supplemented with 10 mM HEPES, pH 7.4, and 0.094 Wünsch units/mL Liberase (Sigma–Aldrich). The liver was dissected carefully, without injuring the capsule, and disrupted using scissors. The dissociated cells were then collected and added to ice-cold high-glucose Dulbecco's modified Eagle's medium (DMEM; Gibco) supplemented with 10% fetal bovine serum (FBS). The cells were pelleted by centrifugation and washed several times. Electroporation was conducted using isolated hepatocytes with a yield of 10–40 × 10^6^ cells and 80% cell viability (measured by trypan blue staining).

### Glycogen staining in hepatocytes

Cells isolated from the liver were stained for glycogen to confirm the successful isolation of hepatocytes (Supplementary Fig. S1). The medium was removed from cultured cells, and the cells were fixed in ice-cold ethanol for 15 min. The cells were incubated in periodic acid 1% in aqueous solution for 5 min and washed. The cells were then incubated in Schiff Reagent for 30 min and washed with water three times over 10 min. Lastly, the cells were mounted in medium and analyzed using a bright-field microscope.

### CRISPR sgRNA design and DNA constructs

The sgRNAs for transfection in mouse and human cells were designed using the Benchling CRISPR Guide RNA Design Tool (benchling.com/crispr). Sequences for different sgRNAs targeting mouse *Hpd* and human *HPD* are shown in Supplementary Table S1. The unmodified and chemically modified sgRNA sequences for the mouse genome are shown in Supplementary Table S2. The sgRNAs transfected into 3T3 cells, Hepa 1-6 cells, and primary mouse and human hepatocytes were obtained from TriLink Biotechnologies or Horizon Discovery Biosciences. The sgRNAs transfected into HEK293 cells were obtained from Integrated DNA Technologies.

The pX330-U6-Chimeric_BB-CBh-hSpCas9 plasmid, a gift from F. Zhang (Addgene plasmid #42230), transformed into DH5α competent cells (Invitrogen). Plasmid DNA was extracted using the Qiagen midi prep protocol. Using standard cloning procedures, annealed oligonucleotides containing guide sequences targeting the mouse *Hpd* locus (Eurofins Genomics) were ligated into the pX330-U6-Chimeric_BB-CBh-hSpCas9 plasmid. Plasmids encoding *Hpd*-aiming CRISPR-Cas9 were evaluated in NIH 3T3 cells.

For constructing the pX330-U6-Chimeric_BB-CBh-hSpCas9Δ_179-595_ plasmid (hereafter Cas9 plasmid DNA), 416 bp gRNA scaffold located between the *Nde*I sites at 179 and 595 was removed by restriction enzyme *Nde*I treatment of the pX330-U6-Chimeric_BB-CBh-hSpCas9 plasmid. Briefly, 2.5 μg of the plasmid DNA was digested with 25 units of *Nde*I (New England Biolabs) at 37°C for 30 min in CutSmart Buffer. The enzyme was inactivated by heating for 20 min at 60°C.

The resulting 8,090 and 416 bp fragments were separated on 1.5% agarose gel containing ethidium bromide. The DNA was visualized under UV light, and the larger 8,090 bp fragment was excised for purification with QIAquick Gel Extraction Kit (Qiagen). Next, 25 ng of the purified linear fragment was ligated with T4 DNA ligase enzyme (New England Biolabs) for 4 h at room temperature and then transformed into DH5α cells. The Cas9 plasmid DNA was isolated using the QIAprep Spin Miniprep kit (Qiagen), and the deletion of the 416 bp fragment was verified using agarose gel electrophoresis.

### Cell culture and electroporation

Mouse NIH 3T3 (Sigma–Aldrich), Hepa 1-6 cells (ATCC), and primary hepatocytes were cultured at 37°C in a humidified incubator with 5% CO_2_ and ambient oxygen levels. Cells were maintained in DMEM (Gibco) supplemented with 10% FBS, 4 mM l-glutamine, and 1 × antibiotic-antimycotic.

Cryopreserved C57BL/6J plateable mouse hepatocytes (product code: MBCP01; Lonza) isolated from 4-month-old male mice were thawed using Rodent and Monkey Cryopreserved Hepatocyte Thawing Medium (Lonza) according to the manufacturer's instructions. Cryopreserved and freshly isolated mouse hepatocytes were maintained in Hepatocyte Plating Medium (Lonza) on six-well Corning Primaria plates. Following cell attachment, the medium was replaced with Hepatocyte Maintenance Medium (Lonza). At 24 h after electroporation, 0.25 mg/mL Corning Matrigel basement membrane matrix was added as an overlay.

Cryopreserved human hepatocytes were obtained from Lonza. Demographic information on the human hepatocyte donors can be found in Supplementary Table S3. According to the manufacturer's instructions, human hepatocytes were thawed using Human Cryopreserved Hepatocyte Thawing Medium (Lonza). Hepatocytes were maintained for 3 days in HCM Hepatocyte Culture Medium Bullet Kit (Lonza). Twenty-four hours after plating, 0.25 mg/mL Corning Matrigel basement membrane matrix was added as an overlay.

The NIH 3T3, Hepa 1-6, HEK293 cells, and human hepatocytes were electroporated with a 4D-Nucleofector X Unit (Lonza). NIH 3T3 cells were electroporated using program EN-158, SG Cell Line 4D-Nucleofector solution (Lonza), and the following conditions: 20 μL SG nucleofection buffer, 2.2 × 10^5^ cells, 0.5 μL of 20 μg/μL sgRNA (TriLink Biotechnologies), and 1.7 μL of 61 μM 3NLS SpCas9 (product code: 1074182; Integrated DNA Technologies). For reactions with Cas9 RNPs, the SpCas9 RNP was incubated with the *Hpd* targeting sgRNA for 20 min at room temperature. Hepa 1-6 and HEK293 cells were electroporated using the SF Cell Line 4D-Nucleofector solution (Lonza) along with the 4D-Nucleofector programs CM-138 and CM-130, respectively.

The Hepa1-6 and HEK293 cell electroporation conditions were as follows: 20 μL SF nucleofection buffer; 1.2 × 10^5^ cells; 0.5 μL of 20 μg/μL sgRNA; and 0.76 μL of 1.32 μg/μL Cas9 pDNA, 1 μL of 1 μg/μL CleanCap Cas9 mRNA (product code: L-7206-1000; TriLink Biotechnologies), 1.7 μL of 61 μM 3NLS SpCas9, or 1.7 uL of 61 μM 3NLS HiFi SpCas9 (product code: 1078728; Integrated DNA Technologies). For cell lines, delivery efficiency was determined by separately electroporating cells with 0.4 μL of 1 μg/μL pmaxGFP (Lonza) and analyzing cells using a Guava Flow Cytometer at 24 h after electroporation (Supplementary Table S4).

Primary human hepatocytes were electroporated using modified procedures described in Zabulica *et al*.^39^ Briefly, hepatocytes were electroporated with P3 Primary Cell 4D-Nucleofector solution (Lonza), program CA-137, and the following electroporation conditions: 100 μL P3 nucleofection buffer, 1.8 μL of 20 μg/μL sgRNA, 5 μL of 61 μM V3 SpCas9 (product code: 1081059; Integrated DNA Technologies) or 5 μL of 61 μM V3 HiFi SpCas9 (product code: 1081061; Integrated DNA Technologies), and 3 μL of 100 μM Electroporation Enhancer (product code: 1075916; Integrated DNA Technologies).

For primary mouse hepatocytes, we first compared different electroporation programs using Lonza 4D and 2b Nucleofectors and observed the highest transfection efficiency using program T-028 on the 2b Nucleofector system (Supplementary Fig. S2). In subsequent experiments, primary mouse hepatocytes were electroporated using Lonza Nucleofector 2b (program T-028), Mouse/Rat Hepatocyte Nucleofector solution (Lonza), and the following electroporation conditions: 100 μL Mouse/Rat Hepatocyte Nucleofector solution, 1.2 × 10^6^ cells, 1.5 μL of 20 μg/μL sgRNA, and 4 μL of 1 μg/μL CleanCap Cas9 mRNA, 4.9 μL of 61 μM 3NLS SpCas9, and 4.9 μL of 61 μM 3NLS HiFi SpCas9.

The delivery efficiency in primary mouse hepatocytes was estimated by electroporating cells with CleanCap eGFP mRNA and analyzing the percentage of green fluorescent protein (GFP)-positive cells using phase and fluorescence microscopy images 24 h after electroporation (Supplementary Table S5). Hepatocytes were stained with trypan blue and counted for cell viability using a hemocytometer.

### Viability and albumin assays

Primary human and mouse hepatocyte viability was measured immediately after electroporation by cell counting after trypan blue staining using a hemocytometer (Supplementary Table S5). In addition, MTT assays (Sigma–Aldrich) were performed on the mouse and human primary hepatocytes 24 h after electroporation, according to the manufacturer's instructions. Briefly, the medium was replaced with fresh culture medium supplemented with 12 mM of MTT stock solution (prepared by adding 1 mL of phosphate-buffered saline to 5 mg of MTT), and the cells were incubated overnight. The produced formazan was dissolved in SDS-HCl solution, incubated for 4 h at 37°C, and the absorbance was measured at 570 nm using a Biotek microplate reader.

For albumin quantification, enzyme immunoassays were carried out for mouse and human albumin using the cell culture mediums collected 1 day post electroporation with the AssayMax mouse/human albumin enzyme-linked immunosorbent assay kits per the manufacturer's instructions (Assaypro, St. Charles, MO). Briefly, 50 μL of standard or sample were added per well and incubated. After washing, 50 μL of biotinylated antibody was added per well and incubated. After washing, 50 μL of SP conjugate was added per well. After incubation, 50 μL of chromogen substrate was added per well and incubated. Lastly, 50 μL of stop solution was added per well, and the absorbances were read immediately at 450 nm.

### Identification and ranking of off-target sites

Potential off-target sites for each sgRNA design were identified using CRISPR-Off-target Sites with Mismatches, Insertions, and Deletions (COSMID).^40^ The CRISPR guide sequence was entered into the program, and a list of related sequences with up to three or fewer base mismatches or sites containing a single base insertion or deletion were considered for potential off-target sites. COSMID generated a list of these sites adjacent to an NRG or NGG PAM site. Sites were then ranked by lowest score and priority given to NGG mismatches over NRG mismatches. We selected 10 off-target sites for deep-sequencing experiments (Supplementary Table S6).

### Measuring allele alterations using Tracking of Indels by Decomposition and deep sequencing

Genomic DNA from electroporated cells was extracted using QuickExtract DNA Extraction Solution (Lucigen) according to the manufacturer's instructions. Primers used for polymerase chain reaction (PCR) amplification of target sites are listed in Supplementary Table S7. PCR was performed with AccuPrime Taq DNA Polymerase (Invitrogen) following the manufacturer's instructions for 35 cycles (94°C for 30 s, 57°C for 30 s, and 68°C for 1 min). Amplicons were purified with the QIAquick PCR Purification kit (Qiagen) or Agencourt AMPure XP beads (Beckman Coulter) and then subjected to Sanger sequencing (Eurofins Genomics). Sanger sequence reads were uploaded to Tracking of Indels by Decomposition (TIDE)^41^ and compared to a control sequence to quantify Cas9-generated insertions/deletions (indels) at the target site.

For next-generation sequencing analysis, we amplified the on- and off-target sites from extracted genomic DNA by two rounds of PCR as described in Lin *et al*.^42^ The first round of PCR added P5 and P7 adaptors to the specific genomic sequence (Supplementary Table S8). The second round of PCR was performed on individual amplicons using primers described previously^42,43^ containing adapter sequences from the first PCR and a unique barcode sequence for samples in the reverse primer (Supplementary Table S9).

PCR reactions for preparing samples for deep sequencing were performed using HotStart Taq DNA Polymerase (New England Biolabs). For the first round of PCR, the reactions were performed with an annealing temperature of 63°C for 35 cycles. The second round of PCR was performed using the PCR product from the first as a template with an annealing temperature of 65°C for 35 cycles. Barcoded amplicons were purified using the QIAquick PCR Purification kit.

The samples were then pooled in equal amounts and resolved on a 1% agarose gel to separate the amplicons from primer dimers. The second PCR amplicon pool was extracted from the gel using the QIAquick Gel Extraction kit (Qiagen). The purified samples were then sequenced by 2 × 250 paired-end sequencing on an Illumina MiSeq platform. Indels were then quantified by a custom script available at https://github.com/piyuranjan/NucleaseIndelActivityScript.

For subsequent generation sequencing experiments in the freshly isolated mouse hepatocytes and H3 of the Hepa 1-6 cells (Supplementary Table S4), on- and off-target sites were amplified using the rhAmpSeq CRISPR Library Kit (product code: 10007318; Integrated DNA Technologies) according to the manufacturer's instructions. Briefly, the rhAmpSeq Mix 1 and rhAmpSeq forward and reverse assay primer pools were designed and synthesized by Integrated DNA Technologies. Genomic DNA was amplified using a thermal cycler with the following conditions: 95°C for 10 min, 10 cycles of 95°C for 15 s and 61°C for 4 min, and 99.5°C for 15 min.

Products of rhAmp PCR products were cleaned up using Sera-Mag Select beads (Cytiva). For the cleanup, 30 μL of beads was added to the PCR products and incubated at room temperature for 10 min. The beads were then collected using a magnetic plate, and the supernatant was discarded. The beads were washed twice with 200 μL of 80% ethanol and then dried at room temperature for 3 min. The beads were then re-suspended in 15 × L of IDTE pH 8.0 (Integrated DNA Technologies) to elute the DNA.

Next, 11 μL of cleaned PCR product was used for the second round of PCR using rhAmpSeq Library Mix 2 and Indexing Primers i5 and i7. The following cycling conditions were used for the second PCR: 95°C for 3 min; 18 cycles of 95°C for 15 s, 60°C for 30 s, and 72°C for 1 min; and 72°C for 1 min. The second-round PCR products were cleaned up using Sera-Mag Select beads. Samples were pooled together, and the library was sequenced by 2 × 250 paired-end sequencing on an Illumina MiSeq platform. FASTQ files from the MiSeq run were uploaded to IDT's rhAmpSeq CRISPR Analysis Tool and merged. Indel quantification was then performed by IDT's data analysis tool, CRISPRAltRations, with the default settings.

### Statistical analysis

The data generated in each experiment were imported into GraphPad Prism v8.0 (GraphPad Software). One-way analysis of variance (ANOVA) and Tukey's multiple comparisons tests were performed to determine the statistical significance of means for comparing on-target indels for Hepa 1-6 experiments and the viability and functionality assays. Two-way ANOVA tests were performed on the deep-sequencing data for the on- and off-target analysis. Unpaired *t*-tests were performed to compare on-target indels between the different samples. *P*-Values <0.05 were considered significant.

## Results

First, we optimized electroporation-mediated delivery of CRISPR-Cas9 targeting mouse *Hpd* to demonstrate proof-of-principle gene editing for an IMD of the liver. We designed four CRISPR guide sequences targeting exon 3 in the *Hpd* locus (Supplementary Fig. S3A) using Benchling (https://benchling.com). The *Streptococcus pyogenes* Cas9 (hereafter Cas9) was used for all experiments described in the study. Plasmid DNA encoding the sgRNA and Cas9 were electroporated into NIH 3T3 cells, and indels were detected via Sanger sequencing and analyzed using TIDE. The sgRNA design labeled Hpd-3 provided the highest on-target indels (Supplementary Fig. S3B) and was used in subsequent experiments.

**FIG. 1.**
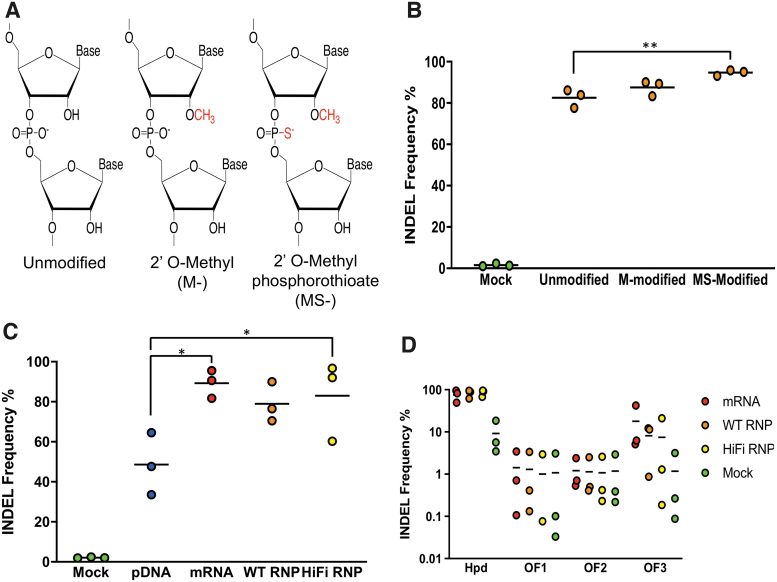
On- and off-target Cas9 activity in electroporated Hepa 1-6 cells. **(A)** Schematic of chemical modifications in the single-guide RNA (sgRNA) structure. **(B)** On-target insertions/deletions (indels) for unmodified and chemically modified synthetic sgRNA co-transfected along with Cas9 protein. **(C)** On-target indels for Cas9 delivered as pDNA, mRNA, wild-type (WT) RNP, and HiFi RNP along with MS-modified sgRNA. **(D)** On- and off-target indels assessed using deep sequencing. Indels were evaluated using Tracking of Indels by Decomposition (TIDE) in **(B)** and **(C)**. *Dots* represent different electroporation experiments, and *horizontal bars* represent the means (*n* = 3). *p < 0.05; **p < 0.01. Mock samples were electroporated with pmaxGFP.

Next, we examined whether chemically modified synthetic sgRNA complexed with Cas9 protein would enhance indels in liver-derived cells. The modifications tested were the addition of 2′-O-methyl (M-sgRNA) or 2′-O-methyl phosphorothioate (MS-sgRNA) linkages to the first and last three consecutive nucleotides on the 5′ and 3′ ends of the sgRNA, respectively (Fig. 1A and Supplementary Table S2). These nucleoside modifications to the sgRNA have been reported to enhance the stability of Cas9 for gene editing in human primary T cells and CD34^+^ HSPCs.^31^

We observed higher gene-editing efficiency in on-target indels for the MS-modified sgRNA than the unmodified sgRNA (Fig. 1B) in Hepa 1-6 cells electroporated with Cas9 RNP complexes (mean 82.6 and 94.7, respectively; *p* = 0.0047). In contrast, we did not see a difference in indels between unmodified and M-modified sgRNA (mean 82.6 and 87.5, respectively; *p* = 0.2482), which indicates that the addition of phosphorothioate combined with the 2′-O-methyl provides enhanced gene editing in Hepa 1-6 cells.

**FIG. 2.**
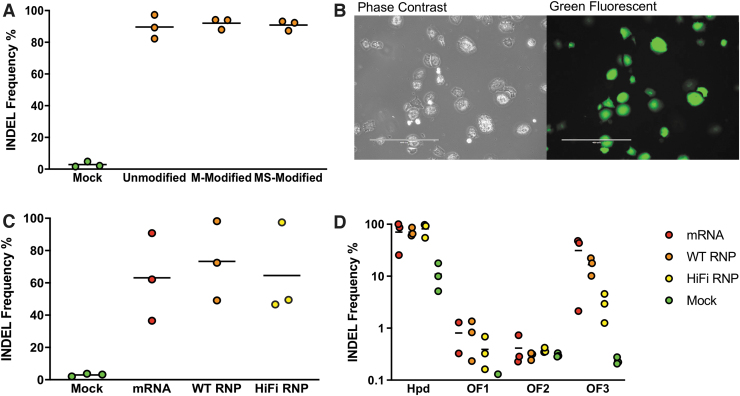
On- and off-target Cas9 activity in electroporated cryopreserved primary mouse hepatocytes. **(A)** On-target indels for unmodified and chemically modified sgRNA electroporated along with Cas9 protein. **(B)** Phase-contrast and fluorescence microscopy image of mouse hepatocytes electroporated with enhanced green fluorescent protein (eGFP) mRNA. The scale bar corresponds to 400 μm. **(C)** Comparison of on-target indels for primary hepatocytes electroporated with Cas9 mRNA, WT RNP, and HiFi RNP. **(D)** Indel frequencies in *Hpd* and three off-target sites in hepatocytes electroporated with Cas9 mRNA, WT RNP, and HiFi RNP. Indels were quantified using TIDE in **(A)**–**(C)** and deep sequencing in **(D)**. *Dots* represent different electroporation experiments, and *horizontal bars* represent the means (*n* = 3). Mock samples were electroporated with eGFP mRNA.

When we repeated this experiment in cryopreserved primary mouse hepatocytes, we observed no significant difference in on-target editing efficiencies between the different modified and unmodified sgRNAs (Fig. 2A). Because the MS-modified sgRNA provided slightly higher indels than the others, we used it in subsequent experiments.

We next evaluated how different forms of Cas9 delivered using electroporation affected the gene-editing efficiency and specificity in Hepa 1-6 cells (Fig. 1C and D). We compared three Cas9 cargos: Cas9 plasmid DNA (pDNA), mRNA, and RNP. The Cas9 plasmid DNA is the pX330-U6-Chimeric_BB-CBh-hSpCas9Δ_179-595_ that had been modified to remove the guide RNA scaffold sequence. The plasmid DNA and mRNA encoding for Cas9 were co-delivered with *Hpd*-aiming sgRNA. For the RNP delivery, Cas9 protein was complexed with *Hpd*-sgRNA before electroporation into cells. The transfection efficiency in Hepa 1-6 cells was >85%, as estimated by electroporating pmaxGFP plasmid DNA (Supplementary Table S4).

In contrast to Cas9 plasmid DNA providing on-target indels of 48.6%, Cas9 mRNA and WT Cas9 RNPs provided higher on-target indels (mean 89.3% and 80.0%, respectively; *p* < 0.9799). We also tested the HiFi Cas9-R691A variant (HiFi Cas9) that reduces off-target activity while maintaining robust on-target gene editing in CD34^+^ HSPCs.^44–46^ Consistent with these previous studies, the on-target indels generated by the HiFi Cas9 RNPs were comparable to Cas9 mRNA and WT Cas9 RNP (Fig. 1C) in Hepa 1-6 cells.

We then assessed the off-target activity of *Hpd*-CRISPR-Cas9 in Hepa 1-6 cells at three COSMID-predicted sites using deep sequencing on an Illumina MiSeq (Fig. 1D). We detected low indels (<2%) at off-target sites OF1 and OF2, whereas high off-target indels (>7.5%) were detected at OF3 in Hepa 1-6 cells electroporated with *Hpd*-CRISPR-Cas9. Electroporation of Cas9 RNP resulted in slightly lower indels at OF3 than Cas9 mRNA, although this was not statistically significant (mean 8.1% and 17.8%, respectively; *p* = 0.6791), while HiFi Cas9 RNP provided similar off-target indels as WT RNP.

Although the number of deep-sequencing reads for the on-target site were low (Supplemental Table S1), the on-target editing efficiency was consistent with the TIDE results obtained using Sanger sequencing. In contrast, we obtained high-sequencing reads for the off-target sites, which is critical for accurately detecting rare off-target editing events.

We evaluated the *Hpd*-targeting CRISPR-Cas9 electroporated as Cas9 mRNA and RNPs in cryopreserved primary mouse hepatocytes. The electroporation efficiency was measured in mouse hepatocytes using fluorescence microscopy 24 h after electroporating eGFP mRNA (Fig. 2B). The mean GFP expression in mouse hepatocytes was 67%, and the cell viability was 31% after electroporation using program T-028 (Supplementary Table S5).

When we applied our electroporation protocol to introduce *Hpd*-CRISPR-Cas9, we observed high levels of on-target indels of >60% for Cas9 mRNA and RNP (Fig. 2C and D) in primary mouse hepatocytes. Consistent with results in Hepa 1-6 cells, we observed comparable on-target indels for the WT and HiFi Cas9 RNPs (Fig. 2C and D) in cryopreserved mouse hepatocytes (mean 73.3% and 64.5%, respectively; *p* = 0.9654). Despite the low deep-sequencing reads at the on-target site (Supplementary Table S2), we obtained similar on-target indels from the deep-sequencing and TIDE analysis.

We observed low indels at OF1 and OF2, while OF3 showed high indels (Fig. 2D) in primary mouse hepatocytes electroporated with Cas9 mRNA (31.2%) and WT RNP (16.7%). Cryopreserved mouse hepatocytes electroporated with HiFi Cas9 RNP showed a fivefold reduction in off-target indels at OF3 compared to WT Cas9 RNP, although this was not statistically significant (mean 2.9% and 16.7%, respectively; *p* = 0.6511).

**FIG. 3.**
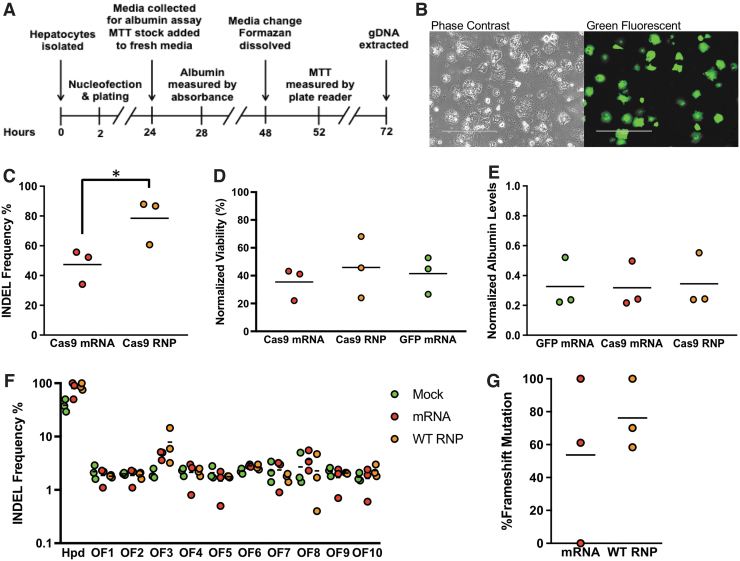
Analysis of on- and off-target Cas9 activity and viability in freshly isolated mouse hepatocytes after electroporation. **(A)** Timeline of experimental procedures. MTT assay and gDNA extraction were performed in separate wells. **(B)** Phase-contrast and fluorescence microscopy image of hepatocytes electroporated with eGFP mRNA. The scale bar corresponds to 400 μm. **(C)** Comparison of on-target indels for isolated mouse hepatocytes treated with Cas9 mRNA or WT Cas9 RNP. **(D)** Viability normalized to untransfected control following electroporation as determined by MTT assay. **(E)** Albumin levels in conditioned media normalized to untransfected control. **(F)** Percentage of indels at on-target and 10 off-target sites measured using deep sequencing. **(G)** Percentage of on-target frameshift mutations in hepatocytes electroporated with Cas9 mRNA and RNP. *Dots* represent different electroporation experiments, and *horizontal bars* represent the means (*n* = 3). *p < 0.05. Mock samples were hepatocytes electroporated with eGFP mRNA.

We next evaluated *Hpd*-CRISPR-Cas9 in mouse hepatocytes freshly isolated from C57BL/6J mice, electroporated within 2 h after collagenase digestion of the liver, and subsequently plated. To determine the effects of electroporation on the hepatocyte viability and functionality, we performed MTT and albumin assays on hepatocytes after electroporation (Fig. 3A). Further, we measured the cell transfection efficiency using microscopy at 24 h after electroporating eGFP mRNA (Fig. 3B).

We observed higher levels of on-target gene editing using the Cas9 RNP than mRNA (mean 78.4% and 47.4%, respectively; *p* = 0.0331) in freshly isolated hepatocytes (Fig. 3C). There was no difference in the MTT and albumin assay results for hepatocytes electroporated with Cas9 mRNA, WT RNP, or GFP mRNA (Fig. 3D and E), which indicates that the CRISPR gene-editing process does not adversely affect the hepatocyte viability or functionality.

For deep-sequencing analysis of Cas9 efficiency and specificity in freshly isolated mouse hepatocytes, we expanded the number of off-target sites to 10. The deep-sequencing data provided low reads for the on-target sites but overall high reads for the off-target sites (Supplemental Table S3). However, the on-target indels from the deep-sequencing data were consistent with the TIDE analysis for the Cas9 mRNA and RNP (Fig. 3F). The high on-target indels detected for the mock (eGFP mRNA) is due to the low sequencing depth.

Overall, the deep-sequencing data confirmed high levels of on-target gene editing in hepatocytes electroporated with Cas9 mRNA (80.1%) and RNP (86.9%). Further, most of these alterations (Fig. 3G) produced by Cas9 mRNA and RNP were frameshift mutations (mean 53.7% and 76.2%, respectively). Consistent with our results in Hepa 1-6 cells and cryopreserved primary mouse hepatocytes, we observed low levels of indels (<3%) at all off-target sites except for OF3. However, the levels of indels at OF3 in freshly isolated mouse hepatocytes were lower than cryopreserved hepatocytes (Fig. 2D). We observed similar indels at OF3 for Cas9 mRNA and RNP (mean 4.6% and 7.9%, respectively; *p* = 0.7499). Our deep-sequencing results suggest that OF3 represents an actual off-target site.

Next, we evaluated the impacts of electroporation to introduce CRISPR-Cas9 into primary human hepatocytes. We tested four different sgRNA designs targeting *HPD* in HEK293 cells (Supplementary Fig. S4A). The four sgRNA designs were electroporated into HEK293 cells alongside Cas9 RNPs using a Lonza 4D nucleofector device, and the on-target indels were quantified using TIDE (Supplementary Fig. S4B). Potential off-target sites for *HPD* were determined using COSMID. After comparing the on-target indels and the number of potential off-targets, we selected sgRNA we labeled *HPD3*, as it had high levels of on-target editing but the fewest potential off-target sites predicted in COSMID (Supplementary Table S10).

**FIG. 4.**
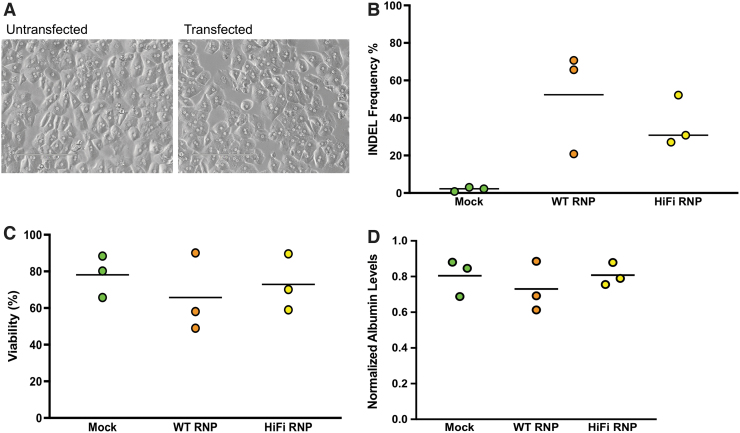
On- and off-target Cas9 activity and viability in electroporated human hepatocytes. **(A)** The first image shows untransfected primary human hepatocytes 24 h after plating. The second image shows plated primary human hepatocytes at 24 h after electroporation. **(B)** On-target indels for human hepatocytes electroporated with WT RNP and HiFi RNP. **(C)** Viability normalized to untransfected control as determined by MTT assay. **(D)** Albumin levels in conditioned media normalized to untransfected control cells. *Dots* represent different electroporation experiments, and *horizontal bars* represent the means (*n* = 3). Mock samples were hepatocytes electroporated with eGFP mRNA.

We electroporated human hepatocytes with WT and HiFi Cas9 RNPs. Electroporated human hepatocytes appeared to have identical cell morphology as the untreated hepatocytes when plated (Fig. 4A). We observed similar levels of on-target editing for the WT RNP and HiFi RNP (mean 52.4% and 36.7%, respectively; *p* = 0.5558) in cryopreserved primary human hepatocytes taken from three different donors (Fig. 4B). The MTT and albumin assays showed high average viability (>60%) for all electroporated cells (Fig. 4C and D), such that there was no significant difference between any groups. These results indicate that the process of CRISPR-Cas9 editing likely does not adversely affect primary human hepatocyte viability.

## Discussion

The present study was performed to (1) determine whether electroporation efficiently delivers CRISPR-Cas9 into primary hepatocytes without adverse effects, and (2) optimize the form of CRISPR components electroporated into hepatocytes. Here, we show that electroporation of CRISPR-Cas9 results in high gene-editing efficiencies in primary mouse and human hepatocytes when delivered as an RNP with overall low levels of off-target alterations.

We tested chemical modifications to the gRNA and found they did not significantly improve gene editing in primary hepatocytes. Our results contrast with the study by Hendel *et al*. that found chemical modifications in sgRNA significantly increased Cas9 editing in primary T cells and CD34+ HSPCs electroporated as RNPs.^31^ The results here indicate that sgRNA used for gene editing in hepatocytes do not require the same chemical protection.

We designed sgRNA targeting the mouse *Hpd* locus to show proof-of-principle CRISPR-Cas9-mediated frameshift mutations to disrupt a therapeutic target gene for an IMD of the liver. Consistent with previous studies,^24,26^ when we electroporated *Hpd*-CRISPR-Cas9 into the Hepa1-6 hepatocellular carcinoma cell line, we observed higher on-target efficiency for Cas9 RNPs and mRNA compared to Cas9 plasmid DNA.

The HiFi Cas9-R691A variant shown to improve gene-editing specificity in electroporated primary human CD34+ HSPCs reduced off-target indels by fivefold in OF3 compared to WT Cas9 RNPs in primary mouse hepatocytes without lowering the on-target activity, although this was not statistically significant. Therefore, HiFi Cas9 electroporated as RNPs slightly enhances gene-editing specificity compared to WT Cas9 in primary hepatocytes.

The study results show that electroporation of CRISPR-Cas9 RNPs in primary mouse and human hepatocytes results in robust gene editing. We also observed similar levels of cell viability (>60%) and albumin production for WT and HiFi Cas9 RNPs in electroporated human primary hepatocytes.

The levels of off-target alterations were only detectable at a single site (OF3) for the mouse *Hpd*-aiming CRISPR-Cas9. The indels in OF3 were slightly reduced using the HiFi Cas9 variant in cryopreserved primary mouse hepatocytes. These results indicate that it is critical to design and screen gRNAs to ensure low to nearly undetectable levels of undesired off-target modifications to enhance the safety of liver-directed gene-editing therapies. We anticipate that the electroporation procedure in primary hepatocytes will have utility for therapeutic gene editing and the development of liver disease models.

Nonviral delivery of CRISPR-Cas9 can address the shortcomings of AAVs while advancing therapeutic applications of liver-directed gene editing. Lipid nanoparticles (LNPs) is the only nonviral delivery approach that has progressed to clinical trials for liver-directed gene-editing therapy (NCT04601051). Like electroporation, LNPs are suitable for delivering CRISPR-Cas9 as an mRNA^47,48^ and RNPs.^49^ In preclinical studies, LNP-mediated delivery of Cas9 mRNA and chemically modified sgRNA targeting *Ttr* in mice resulted in high levels of gene editing (>70%) and >97% knockdown in serum protein levels to correct transthyretin amyloidosis disease indication.^50^ However, toxicity from nanoparticles is a concern that will be evaluated in clinical trials for therapeutic gene editing in patients with hereditary transthyretin amyloidosis.

Another concern with LNPs is that they will likely deliver gene-editing reagents to tissues beyond the targeted tissue. In contrast, nonviral delivery performed *ex vivo* is potentially safer than systemic delivery because the gene editing is only in the intended target cell type and not in the whole organism. An additional advantage of the *ex vivo* approach is the opportunity to (1) maintain target cells in culture until Cas9-derived peptides are no longer expressed on the MHC I surface proteins that can trigger cytotoxic T cells, (2) screen for off-target gene editing, and (3) expand gene-edited hepatocytes using artificial or living bioreactors before transplantation.

The electroporation approach we used in the present study has attractive attributes for CRISPR-mediated therapeutic gene knockdown applications. The studies of Pankowicz *et al*. described the use of *in vivo* hydrodynamically delivered dual CRISPR-Cas9 plasmid DNA encoding a pair of gRNAs to induce the deletion of exons 3 and 4 in *Hpd* to treat HTI in mice.^37^ In our present study, we accomplish frameshift disruptions that lead to gene knockdown using a sgRNA. The dual CRISPR-Cas9 approach for making deletions within the same locus is associated with a higher probability for unwanted off-target effects and genotoxic events compared to using a sgRNA.^51^

Further, our electroporation procedure can be used in autologous hepatocytes from the patient's resected liver followed by transplantation back into the patient to repopulate the liver and is more clinical feasibility compared to hydrodynamic transfection used in Pankowicz *et al*.^37^ for treatment of HTI mice. Electroporation has been applied for *ex vivo* delivery of CRISPR-Cas9 into CD34^+^ HSPCs^25,27,52,53^ and T cells^28,54^ to treat genetic and acquired diseases in clinical trials.

However, the weakness of *ex vivo* gene editing for liver-directed therapy is that only a limited number of disease indications have a natural selective advantage for gene-edited hepatocytes compared to native hepatocytes. Recently, disruption of *Cypor* using a single CRISPR-Cas9 gRNA was shown to enable the enrichment of gene-edited hepatocytes to 50% of the liver mass using acetaminophen,^55^ representing a promising approach selected for a small population of gene-edited hepatocytes for potentially any IMD of the liver.

Another potential therapeutic application of our electroporation approach is for allogenic hepatocyte cell transplantation therapy whereby hepatocytes from healthy donors are engineered using CRISPR-Cas9 to inactivate genes that would avoid graft-versus-host disease against donor hepatocytes analogous to “off-the-shelf” chimeric antigen receptor T cells. Electroporation is attractive for engineering allogeneic “off-the-shelf” hepatocytes because it enables rapid delivery into suspension cells that avoids excessive culturing that can cause loss of cell functionality.

One significant barrier to developing novel therapies for IMDs of the liver is the absence of animal models of human diseases that can be used to conduct studies translated to the clinic. The FRGN chimeric model supports liver humanization and enables repopulation of up to 95% of the liver by human donor hepatocytes.^36,56^ The recent study by Zabulica *et al*. demonstrated the transplantation of patient-derived donor human hepatocytes into FRGN mice to generate a humanized model of ornithine transcarbamylase deficiency.^39^ The weakness of this approach is that the chimeric humanized-liver FRGN mice are immune compromised, which limits the type of experiments that can be conducted.

Alternatively, mouse hepatocytes edited using CRISPR-Cas9 *ex vivo* can be transplanted into the *Fah^–/–^* mice^32,33^ to repopulate the liver with edited hepatocytes for the generation of mouse models of human disease containing a functional immune system. A significant development in this report is the establishment of electroporation procedures for editing primary mouse hepatocytes. We anticipate that hepatocytes edited using our electroporation method can be transplanted into *Fah^–/–^* mice to repopulate the liver.

## Conclusions

In summary, this study demonstrates that electroporation results in high delivery efficiencies in primary mouse and human hepatocytes *ex vivo* as an effective and rapid approach for liver-directed gene editing. We anticipate that our results will stimulate further studies on electroporation and other nonviral methods for *ex vivo* liver-directed gene therapies and the generation of disease models.

## Supplementary Material

Supplemental data
