# Supplementary material for: Electroporation-Mediated Delivery of Cas9 Ribonucleoproteins Results in High Levels of Gene Editing in Primary Hepatocytes
Source: CRISPR J. 2022 Jun 8;5(3):397–409. doi: 10.1089/crispr.2021.0134 (PMC9233506; doi:10.1089/crispr.2021.0134)
Supplement: Supplemental data [file Supp_Data.zip › CottleSupplementalData_rev.docx]

**ELECTROPORATION-MEDIATED DELIVERY OF CAS9 RIBONUCLEOPROTEINS RESULTS IN HIGH LEVELS OF GENE EDITING IN PRIMARY HEPATOCYTES**

**Tanner Rathbone^1^, Ilayda Ates^1^, Lawrence Fernando^1^, Ethan Addlestone^1^, Ciaran M. Lee^2^, Vincent P. Richards^3^, Renee N. Cottle^1*^**

**Supplementary Information**

**Supplementary Figures**

**Supplementary Figure 1.** Glycogen-staining of isolated mouse hepatocytes.

**Supplementary Figure 2.** Comparison of transfection efficiency and viability of different electroporation programs in primary mouse hepatocytes.

**Supplementary Figure 3.** Comparison of Cas9 sgRNA designs targeting *Hpd* in 3T3 cells.

**Supplementary Figure 4.** Comparison of Cas9 sgRNA designs targeting *HPD* in HEK293 cells.

**Supplementary Tables**

**Supplementary Table 1.** Demographic Data of Primary Human Hepatocytes

**Supplementary Table 2.** Electroporation efficiency of Cas9 in Hepa 1-6 and primary mouse hepatocytes.

**Supplementary Table 3.** Sequences of synthetic sgRNA and locations of chemical modifications.

**Supplementary Table 4.** The on-target site within *Hpd*, and three off-target sites generated by COSMID for potential off-target editing.

**Supplementary Table 5.** PCR primers for amplification of *Hpd* for on-target TIDE analysis.

**Supplementary Table 6.** First-step PCR primers for amplification of target sites for Cas9 gene editing.

**Supplementary Table 7.** Primer sequences for second-step PCR for MiSeq.

**Supplementary Table 8.** First-step PCR primers for amplification of target sites for Cas9 gene editing.

**Supplementary Table 9.** Primer sequences for second-step PCR and custom sequencing primers for MiSeq.

**Supplementary Table 10.** Number of potential off-target sites for HPD-targeting sgRNAs.

**
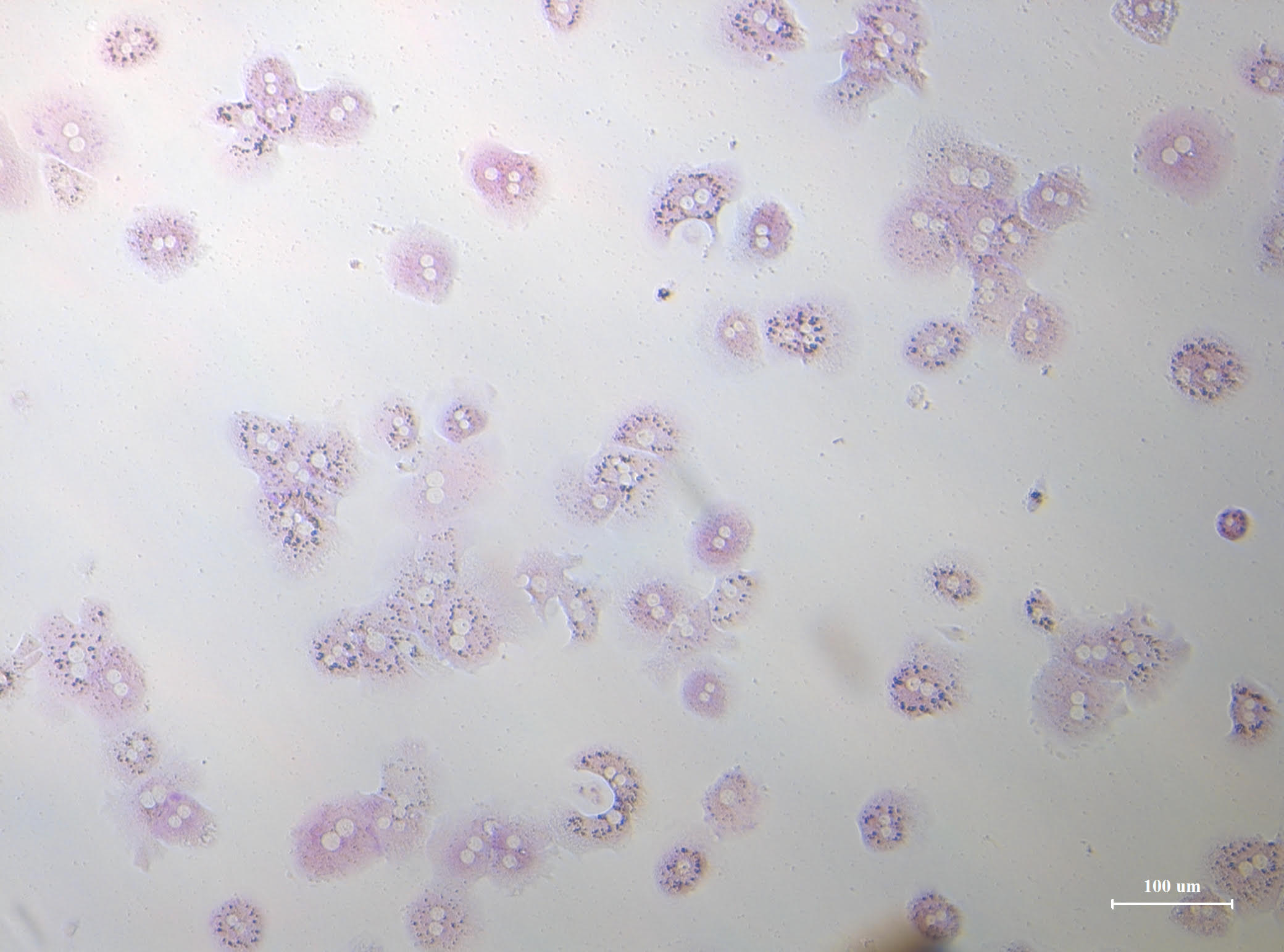

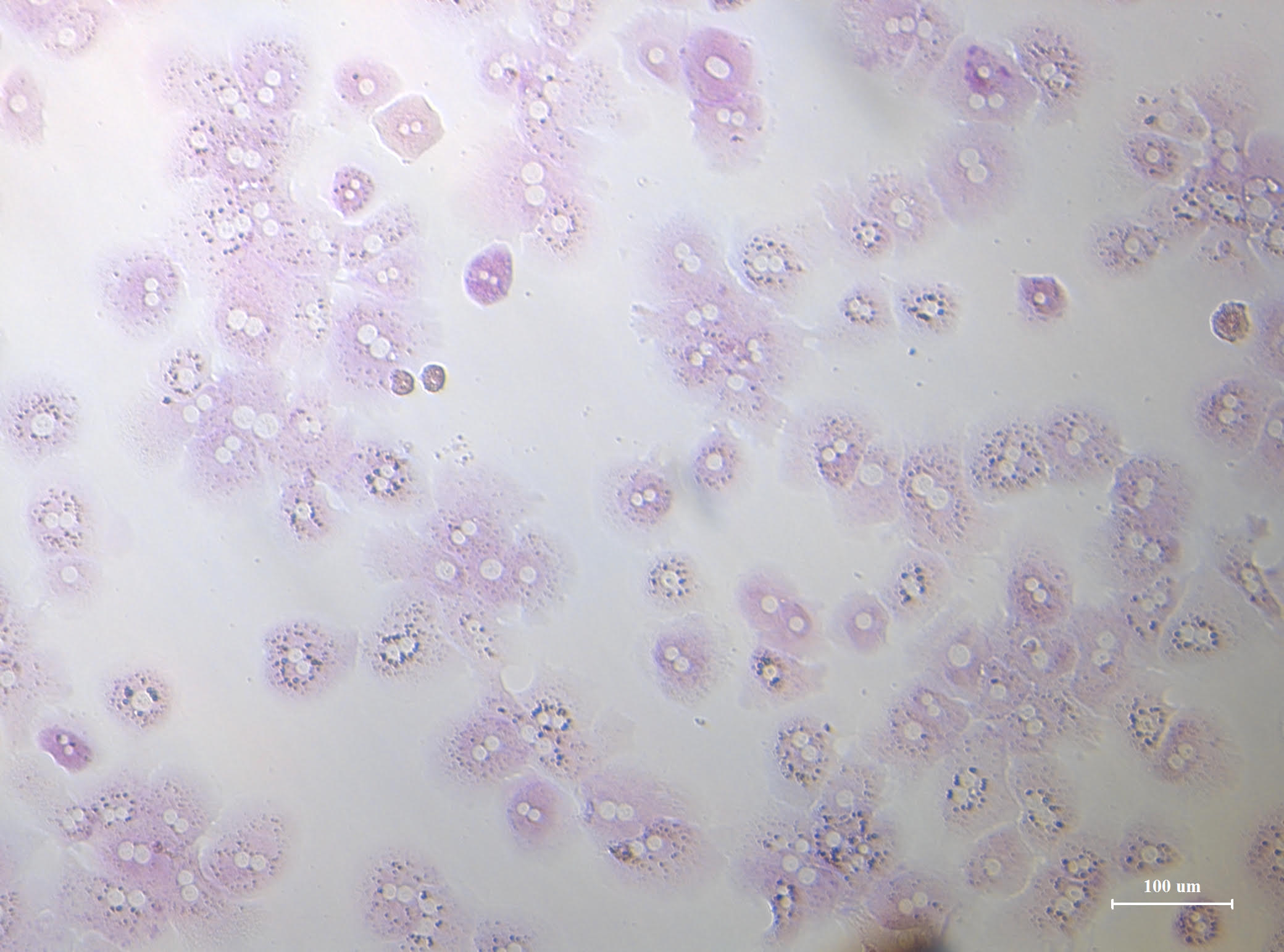
**

**Supplementary Figure 1. Glycogen-staining of isolated mouse hepatocytes.** Cells isolated from C57BL6 mice underwent glycogen-staining to confirm they are hepatocytes. The scale bar corresponds to 100 um.

**
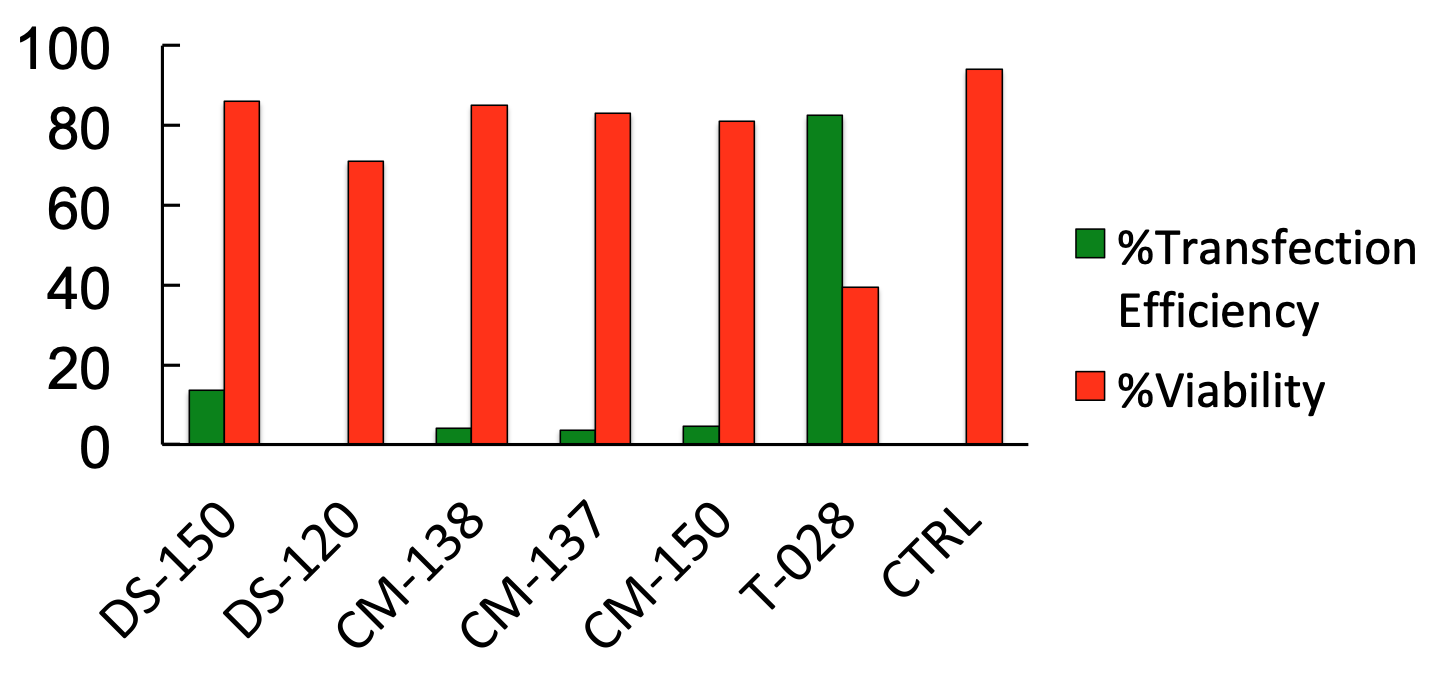
**

**Supplementary Figure 2. Comparison of transfection efficiency and viability of different electroporation programs in primary mouse hepatocytes.** Programs shown were performed with the Lonza 4D Nucleofector device, except for program T-028 which was performed with the Lonza 2b.


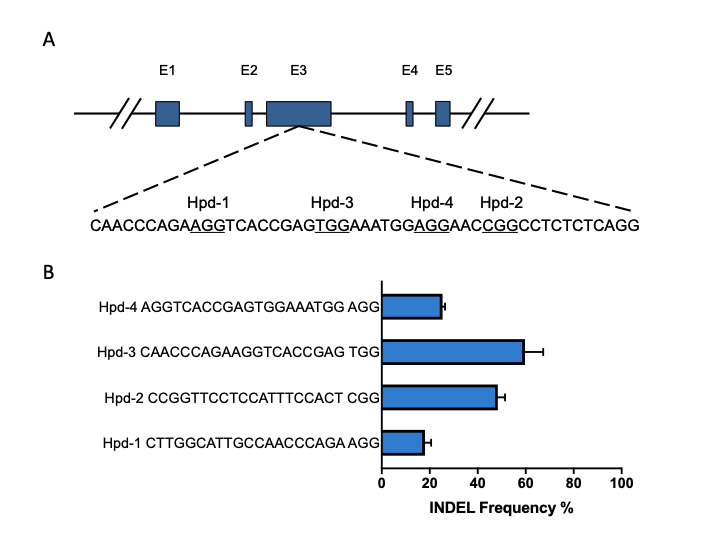


**Supplementary Figure 3. Comparison of Cas9 sgRNA designs targeting *Hpd* in NIH 3T3 cells.** (**A**) Schematic diagram of guide sequences targeting exon 3 of *hpd*. PAM sites are underlined. (**B**) On-target indels in NIH 3T3 cells electroporated with plasmid DNA containing the sgRNA and Cas9 for the different guide sequence designs targeting *Hpd*.

**
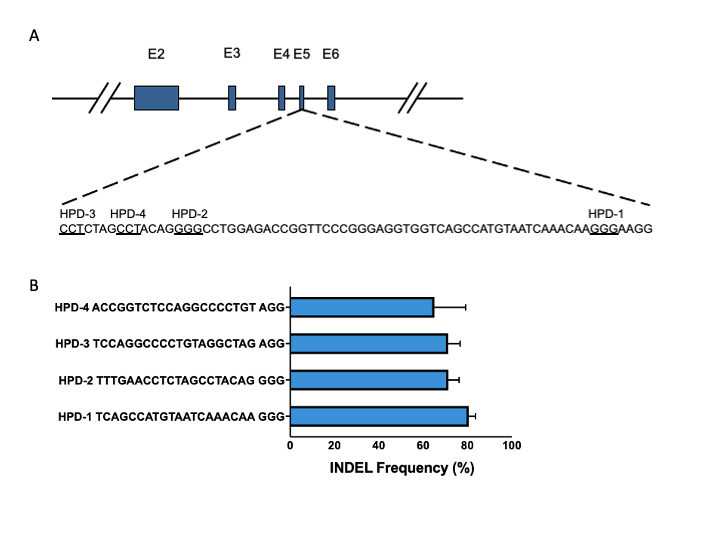
Supplementary Figure 4. Comparison of Cas9 sgRNA designs targeting *HPD* in HEK293 cells.** (**A**) Schematic diagram of guide sequences targeting exon 5 of *HPD*. PAM sites are underlined. (**B**) On-target indels in HEK293 cells nucleofected with the different CRISPR-Cas9 designs target *HPD* delivered as RNPs*.*

**Supplementary Table 1.** **sgRNA sequences for Cas9 comparisons in cultured and primary cells.** (A) Guide sequences targeting exon 3 of *Hpd*. Bolded sequence was used for transfections shown in figures 1-3. (B) Guide sequences for targeting exon 5 of *HPD.* Bolded sequence was used for transfections shown in Figure 4. sgRNA sequences include the PAM-site.

(A)

| sgRNA | Sequence |
| --- | --- |
| Hpd-1 | CTTGGCATTGCCAACCCAGAAGG |
| Hpd-2 | CCGGTTCCTCCATTTCCACTCGG |
| **Hpd-3** | **CAACCCAGAAGGTCACCGAGTGG** |
| Hpd-4 | AGGTCACCGAGTGGAAATGGAGG |

(B)

| sgRNA | Sequence |
| --- | --- |
| HPD-1 | TCAGCCATGTAATCAAACAAGGG |
| HPD-2 | TTTGAACCTCTAGCCTACAGGGG |
| **HPD-3** | **TCCAGGCCCCTGTAGGCTAGAGG** |
| HPD-4 | ACCGGTCTCCAGGCCCCTGTAGG |

**Supplementary Table 2. Sequences of synthetic sgRNA and locations of chemical modifications.** (2’OMe) indicates 2'-O-methyl modifications and (ps) indicates phosphorothioate nucleoside modifications. No additional modifications were made to the sgRNAs other than the ones shown below.

| Modification | Sequence |
| --- | --- |
| Unmodified | 5’ CAA CCC AGA AGG UCA CCG AGG UUU UAG AGC UAG AAA UAG CAA GUU AAA AUA AGG CUA GUC CGU UAU CAA CUU GAA AAA GUG GCA CCG AGU CGG UGC UUU U 3’ |
| M-modified | 5’ 2’OMe (CAA) CCC AGA AGG UCA CCG AGG UUU UAG AGC UAG AAA UAG CAA GUU AAA AUA AGG CUA GUC CGU UAU CAA CUU GAA AAA GUG GCA CCG AGU CGG UGC 2’OMe(UUU) U 3’ |
| MS-modified | 5’ 2’OMe(C(ps)A(ps)A(ps)) CCC AGA AGG UCA CCG AGG UUU UAG AGC UAG AAA UAG CAA GUU AAA AUA AGG CUA GUC CGU UAU CAA CUU GAA AAA GUG GCA CCG AGU CGG UGC 2’OMe(U(ps)U(ps)U(ps)) U 3’ |

**Supplementary Table 3. Demographic information of cryopreserved human hepatocyte donors for electroporation experiments.**

| **Catalog Number** | **Lot Number** | **Donor Age** | **Donor Race** | **Donor Sex** |
| --- | --- | --- | --- | --- |
| HUCPG | HUM191441 | 19 years | Caucasian | Female |
| HUCPG | HUM183001 | 20 years | Caucasian | Female |
| HUCPI | HUM182411 | 5 years | Hispanic | Male |

**Supplementary Table 4. Electroporation efficiency in Hepa 1-6 cells.** H1-H3 refer to Cas9 comparison transfections performed in Hepa 1-6 cells (Figure 1C). Electroporation efficiency was quantified by electroporation of Hepa 1-6 cells with pmaxGFP vector (Lonza) and measuring fluorescence by Millipore Guava easyCyte HT Flow Cytometer at 24-hours post electroporation.

| Experiment | Electroporation Efficiency |
| --- | --- |
| H1 | 85.9% |
| H2 | 96.3% |
| H3 | 91.6% |

**Supplementary Table 5. Electroporation efficiency and viability in cryopreserved mouse hepatocytes following electroporation.** P1-P3 refer to electroporation experiments performed in mouse hepatocytes following thawing (Figure 2C). Electroporation efficiency was quantified by counting the percentage of GFP positive cells using microscopy.

| Experiment | Electroporation Efficiency | Viability |
| --- | --- | --- |
| P1 | 72.7% | 21.1% |
| P2 | 63.6% | 53.1% |
| P3 | 63.4% | 18.8% |

**Supplementary Table 6. The on-target site within *Hpd*, and ten off-target sites generated by COSMID for potential off-target editing.**

| Site Name | Sequence | Location | Gene |
| --- | --- | --- | --- |
| ON | CAACCCAGAAGGTCACCGAGTGG | Chr5:123181875-123181897 | *HPD* |
| OF1 | AATCCCATAAGATCACCGAGAGG | Chr3:3609956-3609978 | *HNF4G* |
| OF2 | AATCCCAGCAGGGCACCGAGGGG | Chr4:117306960-117306982 | *RNF220* |
| OF3 | CAGCCCAGAAGATCAACGAGGGG | Chr18:62977973-62977995 | *NAPG* |
| OF4 | CCACTCAGAATGTCACCGAGAGG | Chr8:125338259-125338281 | Non-coding |
| OF5 | CCACCCAGCAGGGCACCGAGAGG | Chr7:67722981-67723003 | *TTC23* |
| OF6 | AAAGACAGAAGGTCACAGAGAGG | Chr6:66341048-66341070 | Non-coding |
| OF7 | AAAGCCAGAAGGTGCCCGAGTGG | Chr8:11005169-11005191 | *IRS2* |
| OF8 | GAGCCCAGCAGGTCACAGAGAGG | Chr7:73138475-73138497 | Non-coding |
| OF9 | GAAGCCAGAAAGTCACAGAGAGG | Chr7:136293426-136293448 | Non-coding |
| OF10 | TAACTCCGAAGGTCACCAAGAGG | Chr13:43512665-43512687 | Non-coding |

**Supplementary Table 7. PCR primers for amplification of *Hpd* for on-target TIDE analysis.**

**Mouse**

| **FWD** | GGTCACCCATACTGTTCTCACG |
| --- | --- |
| **REV** | \| AGTCCTAGCCTGGCCTGGAT \| \| --- \| \|  \| |

**Human**

| **FWD** | TGTGCAGGCAGCAGTCTATC |
| --- | --- |
| **REV** | \| GAAACTAGGAAGGGCCGTGT \| \| --- \| \|  \| |

**Supplementary Table 8. First-step PCR primers for amplification of target sites for Cas9 gene editing.**

| Forward | Adapter | Gene Specific |
| --- | --- | --- |
| Hpd | TCTACAGTCCGACGATCA | CTCCCCTCGCACTAGCCAAT |
| OF1 | TCTACAGTCCGACGATCA | CCAGTGAGCAGACTCCTCTTTC |
| OF2 | TCTACAGTCCGACGATCA | ATGCAGATGCTTTAGACCCAGTCC |
| OF3 | TCTACAGTCCGACGATCA | TTTGAGTCCTTCGCTTCCCGTAG |
| Reverse | Adapter | Gene Specific |
| Hpd | GACGTGTGCTCTTCCGATC | TAGCCAAAGATGGGAGCAGGG |
| OF1 | GACGTGTGCTCTTCCGATC | CACGAGGGAATTCAGGGTCTTG |
| OF2 | GACGTGTGCTCTTCCGATC | TCCTAGCCTGAGGTGAACTCTCT |
| OF3 | GACGTGTGCTCTTCCGATC | GCAGGGGATTGACCCACAAG |

**Supplementary Table 9. Primer sequences for second-step PCR and custom sequencing primers for MiSeq**. Index sequences are highlighted in red.

| Primer | Sequence |
| --- | --- |
| Index Read | GATCGGAAGAGCACACGTCTGAACTCCAGTCACAT |
| Read 1 | TCTACACGTTCAGAGTTCTACAGTCCGACGATCA |
| Read 2 | TGTGACTGGAGTTCAGACGTGTGCTCTTCCGATC |

| FWD Primer | AATGATACGGCGACCACCGAGATCTACACGTTCAGAGTTCTACAGTCCGACGATCA |
| --- | --- |
| Sample | REV Primer |
| H1A1 | CAAGCAGAAGACGGCATACGAGATAACTCTCGATGTGACTGGAGTTCAGACGTGTGCTCTTCCGATC |
| H1B1 | CAAGCAGAAGACGGCATACGAGATACTATGTCATGTGACTGGAGTTCAGACGTGTGCTCTTCCGATC |
| H1C1 | CAAGCAGAAGACGGCATACGAGATAGTAGCGTATGTGACTGGAGTTCAGACGTGTGCTCTTCCGATC |
| H1D1 | CAAGCAGAAGACGGCATACGAGATCAGTGAGTATGTGACTGGAGTTCAGACGTGTGCTCTTCCGATC |
| H1E1 | CAAGCAGAAGACGGCATACGAGATCGTACTCAATGTGACTGGAGTTCAGACGTGTGCTCTTCCGATC |
| H1F1 | CAAGCAGAAGACGGCATACGAGATCTACGCAGATGTGACTGGAGTTCAGACGTGTGCTCTTCCGATC |
| H1G1 | CAAGCAGAAGACGGCATACGAGATGGAGACTAATGTGACTGGAGTTCAGACGTGTGCTCTTCCGATC |
| H1H1 | CAAGCAGAAGACGGCATACGAGATGTCGCTCGATGTGACTGGAGTTCAGACGTGTGCTCTTCCGATC |
| H1Mock | CAAGCAGAAGACGGCATACGAGATGTCGTAGTATGTGACTGGAGTTCAGACGTGTGCTCTTCCGATC |
| H2A1 | CAAGCAGAAGACGGCATACGAGATTAGCAGACATGTGACTGGAGTTCAGACGTGTGCTCTTCCGATC |
| H2B1 | CAAGCAGAAGACGGCATACGAGATTCATAGACATGTGACTGGAGTTCAGACGTGTGCTCTTCCGATC |
| H2C1 | CAAGCAGAAGACGGCATACGAGATTCGCTATAATGTGACTGGAGTTCAGACGTGTGCTCTTCCGATC |
| H2D1 | CAAGCAGAAGACGGCATACGAGATAAGTCGAGATGTGACTGGAGTTCAGACGTGTGCTCTTCCGATC |
| H2E1 | CAAGCAGAAGACGGCATACGAGATATACTTCGATGTGACTGGAGTTCAGACGTGTGCTCTTCCGATC |
| H2F1 | CAAGCAGAAGACGGCATACGAGATAGCTGCTAATGTGACTGGAGTTCAGACGTGTGCTCTTCCGATC |
| H2H1 | CAAGCAGAAGACGGCATACGAGATCATAGAGAATGTGACTGGAGTTCAGACGTGTGCTCTTCCGATC |
| H2H1 | CAAGCAGAAGACGGCATACGAGATCGTAGATCATGTGACTGGAGTTCAGACGTGTGCTCTTCCGATC |
| H2Mock | CAAGCAGAAGACGGCATACGAGATCTCGTTACATGTGACTGGAGTTCAGACGTGTGCTCTTCCGATC |
| Hepa1-6 Untr | CAAGCAGAAGACGGCATACGAGATGCGCACGTATGTGACTGGAGTTCAGACGTGTGCTCTTCCGATC |
| P1A1 | CAAGCAGAAGACGGCATACGAGATGGTACTATATGTGACTGGAGTTCAGACGTGTGCTCTTCCGATC |
| P1A2 | CAAGCAGAAGACGGCATACGAGATGTATACGCATGTGACTGGAGTTCAGACGTGTGCTCTTCCGATC |
| P1B1 | CAAGCAGAAGACGGCATACGAGATTACGAGCAATGTGACTGGAGTTCAGACGTGTGCTCTTCCGATC |
| P1B2 | CAAGCAGAAGACGGCATACGAGATTCAGCGTTATGTGACTGGAGTTCAGACGTGTGCTCTTCCGATC |
| P1C1 | CAAGCAGAAGACGGCATACGAGATTCGCTACGATGTGACTGGAGTTCAGACGTGTGCTCTTCCGATC |
| P1C2 | CAAGCAGAAGACGGCATACGAGATACCTACTGATGTGACTGGAGTTCAGACGTGTGCTCTTCCGATC |
| P1Mock | CAAGCAGAAGACGGCATACGAGATAGCGCTATATGTGACTGGAGTTCAGACGTGTGCTCTTCCGATC |
| P2A1 | CAAGCAGAAGACGGCATACGAGATAGTCTAGAATGTGACTGGAGTTCAGACGTGTGCTCTTCCGATC |
| P2A2 | CAAGCAGAAGACGGCATACGAGATCATGAGGAATGTGACTGGAGTTCAGACGTGTGCTCTTCCGATC |
| P2B1 | CAAGCAGAAGACGGCATACGAGATCTAGCTCGATGTGACTGGAGTTCAGACGTGTGCTCTTCCGATC |
| P2B2 | CAAGCAGAAGACGGCATACGAGATCTCTAGAGATGTGACTGGAGTTCAGACGTGTGCTCTTCCGATC |
| P2C1 | CAAGCAGAAGACGGCATACGAGATGAGCTCATATGTGACTGGAGTTCAGACGTGTGCTCTTCCGATC |
| P2C2 | CAAGCAGAAGACGGCATACGAGATGGTATGCTATGTGACTGGAGTTCAGACGTGTGCTCTTCCGATC |
| P2Mock | CAAGCAGAAGACGGCATACGAGATGTATGACGATGTGACTGGAGTTCAGACGTGTGCTCTTCCGATC |
| P3A1 | CAAGCAGAAGACGGCATACGAGATTAGACTGAATGTGACTGGAGTTCAGACGTGTGCTCTTCCGATC |
| P3A2 | CAAGCAGAAGACGGCATACGAGATTCACGATGATGTGACTGGAGTTCAGACGTGTGCTCTTCCGATC |
| P3B1 | CAAGCAGAAGACGGCATACGAGATTCGAGCTCATGTGACTGGAGTTCAGACGTGTGCTCTTCCGATC |
| P3B2 | CAAGCAGAAGACGGCATACGAGATACCTAGTAATGTGACTGGAGTTCAGACGTGTGCTCTTCCGATC |
| P3C1 | CAAGCAGAAGACGGCATACGAGATACGTACGTATGTGACTGGAGTTCAGACGTGTGCTCTTCCGATC |
| P3C2 | CAAGCAGAAGACGGCATACGAGATATATCGCGATGTGACTGGAGTTCAGACGTGTGCTCTTCCGATC |
| P3Mock | CAAGCAGAAGACGGCATACGAGATCACGATAGATGTGACTGGAGTTCAGACGTGTGCTCTTCCGATC |
| Primary Untr | CAAGCAGAAGACGGCATACGAGATCGTATCGCATGTGACTGGAGTTCAGACGTGTGCTCTTCCGATC |
| IB1 | CAAGCAGAAGACGGCATACGAGATCTGCGACTATGTGACTGGAGTTCAGACGTGTGCTCTTCCGATC |
| IB2 | CAAGCAGAAGACGGCATACGAGATGCTGTAACATGTGACTGGAGTTCAGACGTGTGCTCTTCCGATC |
| IC1 | CAAGCAGAAGACGGCATACGAGATGGACGTTAATGTGACTGGAGTTCAGACGTGTGCTCTTCCGATC |
| IC2 | CAAGCAGAAGACGGCATACGAGATGGTCGTAGATGTGACTGGAGTTCAGACGTGTGCTCTTCCGATC |
| Isolated Untr | CAAGCAGAAGACGGCATACGAGATTAAGTCTCATGTGACTGGAGTTCAGACGTGTGCTCTTCCGATC |

**Supplementary Table 10.** **Number of potential off-target sites for HPD-targeting sgRNAs**. Off-target sites for the HPD-targeting sgRNAs shown in Supplementary Fig. 4B were identified using COSMID. Conditions for identifying off-target sites included sites with up to three mismatches a 1-bp insertion, or a 1-bp deletion in the sequence, and up to two mismatches in the PAM sequence.

| Sequence Name | sgRNA Sequence | Query Sequence | Total # of Off-Target Sites |
| --- | --- | --- | --- |
| HPD1 | TCAGCCATGTAATCAAACAA | NCAGCCATGTAATCAAACAANRG | 474 |
| HPD2 | TTTGAACCTCTAGCCTACAG | NTTGAACCTCTAGCCTACAGNRG | 333 |
| HPD3 | TCCAGGCCCCTGTAGGCTAG | NCCAGGCCCCTGTAGGCTAGNRG | 129 |
| HPD4 | ACCGGTCTCCAGGCCCCTGT | NCCGGTCTCCAGGCCCCTGTNRG | 170 |
